# Enhancing transcranial direct current stimulation via motor imagery and kinesthetic illusion: crossing internal and external tools

**DOI:** 10.1186/s12984-016-0156-3

**Published:** 2016-06-01

**Authors:** Florian Bodranghien, Mario Manto, Florent Lebon

**Affiliations:** Unité d’Etude du Mouvement, Laboratoire de Neurologie Expérimentale, ULB, Brussels, Belgium; Service des Neurosciences, Université de Mons, Mons, Belgium; Laboratoire INSERM U1093 Cognition, Action et Plasticité Sensorimotrice, Université de Bourgogne Franche-Comté, Dijon, France; UFR STAPS, Université de Bourgogne Franche-Comté, Dijon, France; UEM, FNRS-ULB, 808 Route de Lennik, 1070 Bruxelles, Belgium

**Keywords:** Direct current stimulation, Motor imagery, Kinesthetic illusion, Excitability, Rehabilitation

## Abstract

**Background:**

Transcranial direct current stimulation is a safe technique which is now part of the therapeutic armamentarium for the neuromodulation of motor functions and cognitive operations. It is currently considered that tDCS is an intervention that might promote functional recovery after a lesion in the central nervous system, thus reducing long-term disability and associated socio-economic burden.

**Discussion:**

A recent study shows that kinesthetic illusion and motor imagery prolong the effects of tDCS on corticospinal excitability, overcoming one of the limitations of this intervention.

**Conclusion:**

Because changes in excitability anticipate changes in structural plasticity in the CNS, this interesting multi-modal approach might very soon find applications in neurorehabilitation.

Transcranial direct current stimulation (tDCS) is a non-invasive brain stimulation technique, which consists of delivering a low (usually between 1 and 2.5 mA) constant current between two electrodes positioned on the skull. Depending on the polarity chosen, tDCS is either cathodal or anodal. In the first set up, the cathodal electrode is positioned over the target brain area and lowers the underlying neurons’ action potential firing rate, therefore decreasing neural excitability. In the second set up, the anodal electrode is positioned over the target area and increases the action potential firing rate, therefore inducing a hyperexcitability [3]. The so-called after-effects depend on the set up, the duration and the intensity of the stimulation [11]. For anodal DCS, the intra-DCS effects are much less prominent when compared to the after-effects. However, for cathodal DCS, the intra-DCS effects and the after-effects are nearly similar [16]. One of the limitations of DCS is related to the fact that the after-effects will disappear after a few hours, hence the importance of repeating the application in the following days or weeks. Despite this limitation, due to its ease of use, its safety and its low cost, tDCS is growingly applied to modulate central nervous system (CNS) excitability in fundamental research [19] as well as in trials involving human healthy volunteers or patients [2]. In particular, there is a great hope that tDCS will be helpful not only for the acute management or rehabilitation of motor neurological disorders, but even beyond, for instance in cognitive or psychiatric disorders, although optimizations of the stimulation parameters are still clearly required [7, 13].

While tDCS artificially modulates neural excitability, other methods use afferent inputs or even internal representations of movement to induce cortical plasticity. Kinesthetic illusion (KI) is based on various sources of sensory stimuli to activate cerebral networks. For example, a hand motion video, if positioned appropriately, induces the subjective feeling of movement in one’s hand [9]. It is usually assumed that the right cerebral hemisphere plays a critical role in the conscious experience of the body. This is confirmed by recent fMRI studies demonstrating that kinesthetic illusory movement activates the right frontoparietal regions [1]. Considered globally, investigations of proprioceptive bodily illusions show a hierarchy of three brain systems: the motor network processing afferent inputs from skeletal muscles in order to build kinematic/dynamic postural models of limbs, parietal regions integrating the information across different coordinate systems in order to maintain the adaptability of the body representation, and the right inferior fronto-parietal network recruited when bodily illusions are concerned [14]. One of the potential clinical applications of KIs in the coming years is the management of painful states [4].

Interestingly, motor imagery (MI), the internal representation of movement without concomitant contraction, induces similar brain activation as the actual motor performance [17]. These neuroanatomical correlates legitimate the benefits of MI-based mental practice on motor learning [8]. MI-based motor learning impacts on brain networks, especially the functional connectivity of the default mode network [5]. It is not surprising that MI is now used with the goal of improving motor skills, self-motion perception or facilitating decision-making [5, 15]. Its potential for clinical prevention or rehabilitation treatment becomes prominent.

As pointed out earlier, one of the practical issues with tDCS is its effect over a limited period of time. Any method that could increase the duration of the effects of tDCS would be practically very useful. The article by Kaneko et al. presents novel findings on how to prolong the after-effects of anodal tDCS (tDCSa) [10]. The authors included 21 healthy volunteers and assessed the combination of tDCSa with KI and/or MI on corticospinal excitability assessed by transcranial magnetic stimulation (TMS). Subjects underwent four different scenarios in a randomized order: 1) tDCSa alone, 2) tDCSa combined with KI, 3) tDCSa combined with MI and 4) tDCSa combined with KI and MI. In a control experiment, some subjects were tested with sham tDCSa (a placebo equivalent for the tDCS technique) combined with KI and MI. During the tDCSa intervention, the anodal and cathodal electrodes were positioned over the motor area for the first dorsal interosseous and above the contralateral orbit, respectively. KI was induced by the visualization of finger movements, through a screen placed over the right arm of participants. During MI, participants were instructed to feel the muscle contraction normally induced by actual finger movement (kinesthetic modality). The authors positioned a figure-of-eight coil of the cortical representation of the left first dorsal interosseous to evoke motor-evoked potentials (MEPs) in the targeted muscle. They recorded MEPs before and right after the intervention, as well as 30 and 60 min after the intervention to test the after-effects. The authors found a prolonged increase of corticospinal excitability (i.e., a greater after-effect) up to 30 min after the intervention combining tDCSa with KI and MI, in comparison to other interventions. The absence of modulation after sham tDCS combined with KI and MI highlighted the priming effect of tDCSa on cortical modulation for an acute session. The authors suggested that Hebb’s rule could partly explain these observations. Simultaneous stimulation of the CNS would induce a pronounced increase in terms of synaptic efficacy and strength of the system [6]. The authors suggest that the activation of the cerebral cortex and in particular the corticospinal tract during MI and KI may have boosted the effect of tDCSa. This would support the recent findings by Saimpont et al. [18] who found that MI training coupled with tDCSa greatly improved hand motor function in healthy subjects.

In conclusion, the combination of tDCS with KI and MI is now an opportunity given to the rehabilitation teams to obtain a sustained increase in the excitability of the motor cortex. Because the modulation of the motor cortex excitability is considered as an early change before occurrence of structural plasticity [12], crossing MI, KI and tDCS might be of great interest for studies on learning and brain recovery. Enhancing cognitive and motor functions in people with physical disabilities remains a major goal for researchers and rehabilitation specialists. It is now important to further assess the effects of such combination tDCS/KI/MI in larger groups of control subjects and in clinical trials.
